# Boat noise alters individual behaviors but not communication between partners in a fish-shrimp mutualism

**DOI:** 10.1093/beheco/araf110

**Published:** 2025-09-27

**Authors:** Jack L Manera, Jake M Martin, Maria M Palacios, Rachel T Mason, Mark I McCormick, Bob B M Wong

**Affiliations:** School of Biological Sciences, Monash University, 25 Rainforest Walk, Clayton, Victoria 3800, Australia; School of Biological Sciences, Monash University, 25 Rainforest Walk, Clayton, Victoria 3800, Australia; School of Life and Environmental Sciences, Deakin University, 75 Pigdons Road, Waurn Ponds, Victoria 3216, Australia; Department of Wildlife, Fish, and Environmental Studies, Swedish University of Agricultural Sciences, Umeå 901 83, Sweden; Centre for Nature Positive Solutions, School of Science, RMIT University, GPO Box 2476, Melbourne, Victoria 3000, Australia; School of Life and Environmental Sciences, Deakin University, 75 Pigdons Road, Waurn Ponds, Victoria 3216, Australia; Coastal Marine Field Station, School of Science, University of Waikato, 58 Cross Road, Sulphur Point, Tauranga 3110, New Zealand; School of Biological Sciences, Monash University, 25 Rainforest Walk, Clayton, Victoria 3800, Australia

**Keywords:** *Amblyeleotris steinitzi*, animal behavior, field study, interspecific interactions, noise pollution, ocean noise

## Abstract

Persistent noise pollution produced by boat traffic is reshaping marine soundscapes globally. Despite growing ecological concern, most studies to date have focused on individual-level effects under laboratory conditions, leaving major gaps in our understanding of how boat noise shapes species interactions in the wild. Using field-based behavioral assays, we investigate how boat noise from different engine types (4-stroke and 2-stroke) affects the mutualistic partnership between Steinitz's goby (*Amblyeleotris steinitzi*) and snapping shrimp (*Alpheus* spp.). Across 123 partnerships, we recorded behavioral responses before, during, and after noise exposure. Gobies increased burrow use during 4-stroke boat noise exposure, while shrimp responded stronger to 2-stroke noise—reflecting taxon-specific sensitivities to different noise spectra. Despite these shifts, tactile partner communication was not affected by boat noise. These findings highlight divergent vulnerabilities between species tied to different engine acoustics and emphasize the need for targeted research to inform strategies for mitigating marine noise pollution.

## Teaser

Boat noise disrupts how gobies and shrimp share their burrows, with each species responding differently depending on engine type. While gobies perceived the noise from 4-stroke powered boats as a greater threat, shrimp responded stronger to 2-stroke noise. Despite these shifts, their tactile communication—key to their mutualism—was robust to noise exposure. Our study highlights that engine choice can shape species-specific responses, informing better management of marine noise pollution.

## Introduction

Human-generated noise is altering acoustic environments around the world. This issue is particularly pronounced in aquatic settings (Kunc et al. 2016), due to the distinct acoustic properties of water, where sound waves travel faster, further, and with greater intensity (Larsen and Radford 2018). Boat noise is one of the most prevalent forms of marine noise pollution (Hildebrand 2009). Large vessels traveling along global shipping lanes and smaller boats navigating coastlines and river systems produce a constant, and growing, source of noise pollution (Jalkanen et al. 2022; Wilson et al. 2022). Consequently, there are very few aquatic ecosystems that are not exposed to some form of boat noise (Duarte et al. 2021). The noise produced by boats typically falls within the 10 to 5,000 Hz frequency range (Hildebrand 2009), which coincides with the hearing ranges of many aquatic wildlife, including fish (50 to 1,500 Hz; Popper and Fay 2011) and invertebrates (eg snapping shrimp, 40 to 1,500 Hz; Dinh and Radford 2021), making such taxa especially susceptible to boat noise pollution.

The bulk of research to date, has focused on the effects of boat noise on individual species in laboratory settings, however, there is growing recognition of the need for ecologically relevant, field-based assessments. In confined environments, such as experimental aquaria, sound pressure and particle motion—the two fundamental components of sound waves—behave very differently compared to open bodies of water (Slabbekoorn 2016). This discrepancy makes field studies essential for replicating environmentally realistic sound conditions. Importantly, field assessments also provide the opportunity to examine how noise influences species within the broader context of the natural ecosystems they inhabit. Organisms do not exist in isolation; instead, their survival and fitness are often shaped by ecological interactions, such as predation, parasitism, competition, and mutualism (Brown et al. 2001). These interactions can be disrupted (Nedelec et al. 2017a), or enhanced (Fernandez-Declerck et al. 2023), by noise pollution exposure, which has consequences for the stability and functioning of entire ecosystems (Francis et al. 2009). Thus, investigating the effects of boat noise on species interactions—rather than just on individual taxa—is crucial to gain a comprehensive understanding of the range of potential impacts of noise pollution.

**Fig. 1.**
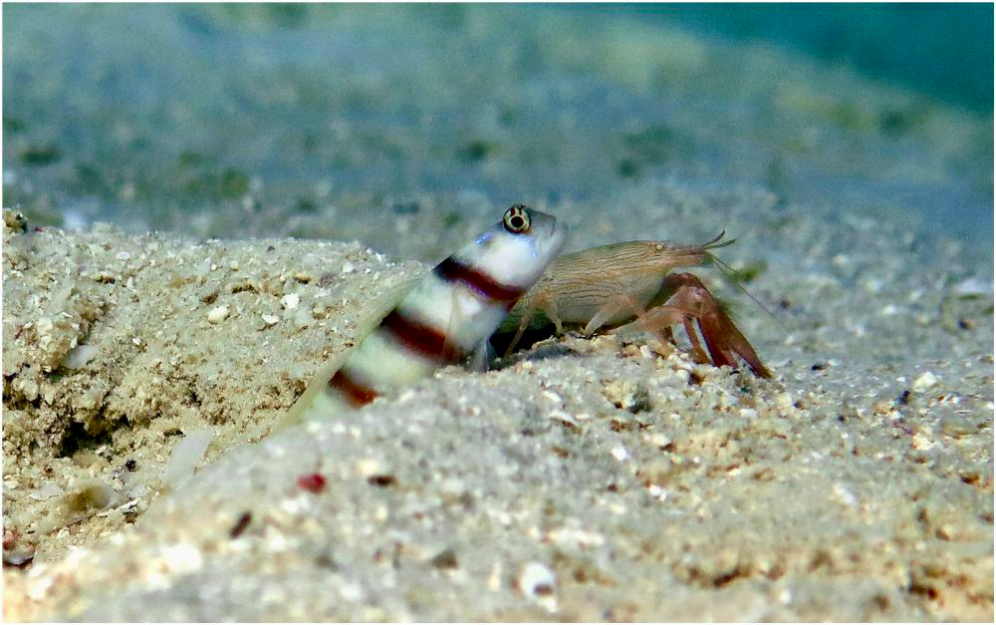
Example of the mutualism between a Steinitz's goby (*Amblyeleotris steinitzi*) and a snapping shrimp (*Alpheus mannarensis*), at lizard island lagoon. Photographed by MMP.

Accordingly, we set out to investigate the impacts of boat noise pollution on the behavioral interactions between two susceptible marine taxa: fish and crustaceans. To do so, we capitalized on the iconic and well-characterized mutualistic relationship between prawn gobies and burrowing shrimp, focusing specifically on Steinitz's goby (*Amblyeleotris steinitzi*) and snapping shrimp (*Alpheus* spp.; Fig. 1). In these goby-shrimp mutualisms, which can involve more than one individual shrimp and/or goby, the shrimp excavate and maintain a shared burrow. In return, the goby serves as a sentinel, performing vigilance behaviors outside the burrow to detect, and communicate, potential threats to the shrimp. We hypothesized that boat noise exposure would disturb the mutualism of these two species by independently altering perceived risk, leading to shifts in behavior, and potentially disrupting their communication. To test this, we used outboard engines on small dinghies representative of those typically operating over shallow reefs. Our study included both 4-stroke and 2-stroke engines, as these are the most widely used in vessels and have previously been shown to impact fish and invertebrates (Nedelec et al. 2014, 2017b; McCormick et al. 2018). Although the acoustic spectra of both engines are broadly similar, 2-stroke motors exhibit lower acoustic complexity and generally higher intensity across most key metrics (eg, higher root-mean-square levels, higher peak energy, larger 90% energy envelope, and higher consistency; McCormick et al. 2018, 2019). Consequently, we predicted that noise from 2-stroke engines would have a greater impact on the behavior of gobies and shrimp (sensu Jain-Schlaepfer et al. 2018; McCormick et al. 2018).

## Materials and methods

Methods are reported following “Method Reporting with Initials for Transparency” (MeRIT), to further clarify contributor roles for reproducibility and replicability (Nakagawa et al. 2023).

### Study site

**Fig. 2.**
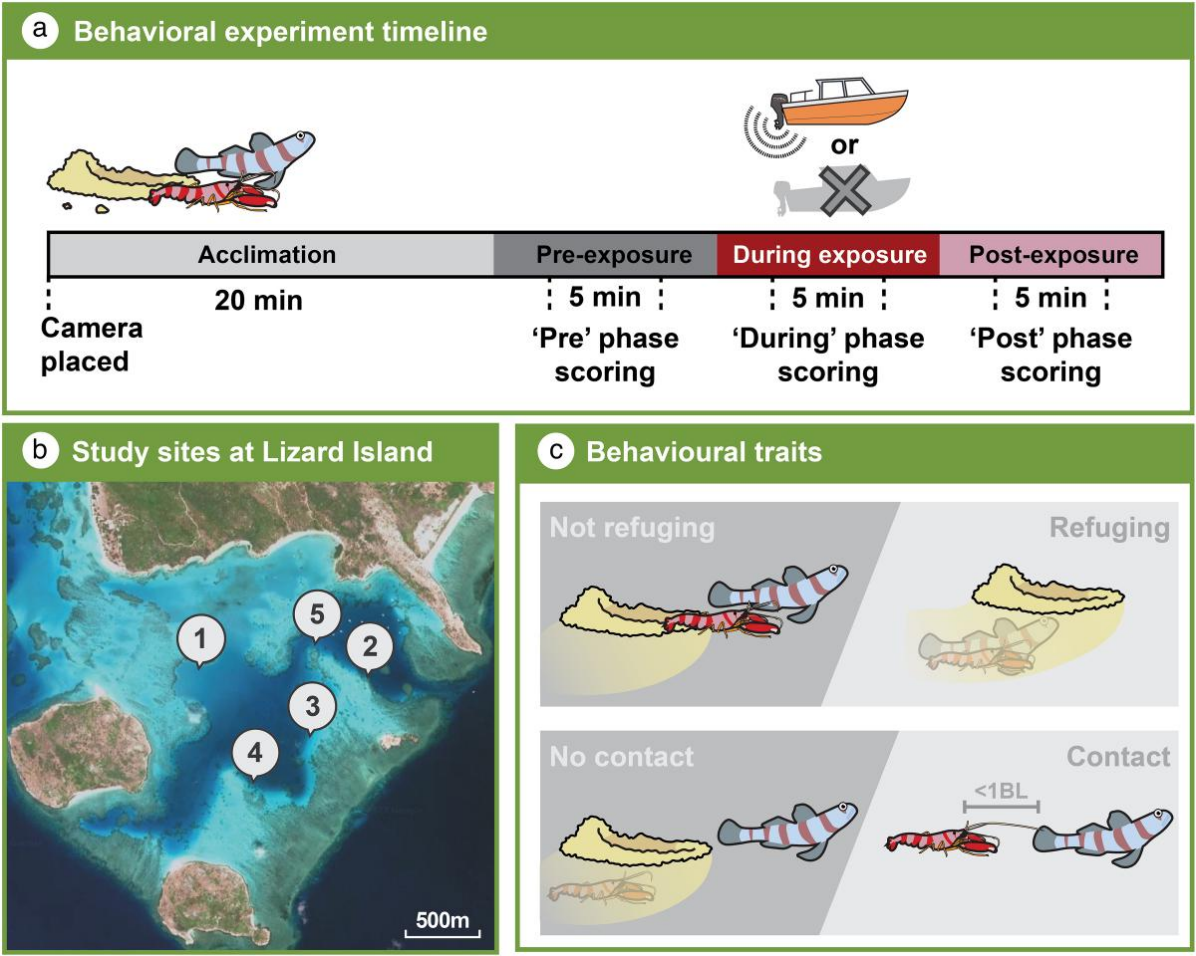
Experimental setup and site map for assessing the effects of boat noise on goby and shrimp behavior. (a) The distinct phases of each trial, including a 20 min acclimation period, followed by 10 min pre-, during-, and post-noise treatment phases, with a 5 min observation window for behavioral recordings in the middle of each phase. (b) A map of the sample site locations within the Lizard Island lagoon, Great Barrier Reef, Australia (14°41′9″S, 145°27′21″E; see Table S1 for site-specific sample sizes). (c) The behavioral measurements including refuge use and tactile communication, which was assessed by the physical proximity of a shrimp to a goby.

This study was performed between 25 October and 25 November 2017 at Lizard Island (14°41′9″S, 145°27′21″E) on the Great Barrier Reef, Australia. Within the lagoon, 5 different sites were identified as sampling locations (see Fig. 2). This lagoon was selected as it represents a section of the Great Barrier Reef lagoonal basin with relatively low vessel traffic (McCormick et al. 2018). Each site had a large population of Steinitz's gobies with burrows located on soft sediments at 2 to 3 m water depth and 1 to 2 m from the reef edge. The experimental design was conceived by MMP, BBMW, and MIM.

### Boat noise treatments

The vessels used for noise pollution treatments were 5 m long aluminum hull dinghies, with either a 30 horsepower 4-stroke outboard motor (Suzuki DF30A) or a 30 horsepower 2-stroke outboard motor (Suzuki DT30; *n* = 3 boats per engine type), with identical hull design. Both 4-stroke and 2-stroke motorboats were used as distinct treatments in this study because of the different sound characteristics of the 2 motors, and the potential for different effects on wildlife (Jain-Schlaepfer et al. 2018; McCormick et al. 2018, 2019). The 4-stroke and 2-stroke boats deployed in the current study are identical to those used in previously published research where measurements were made at similar locations on the same reef under approximately the same weather conditions; as a result, their acoustic properties have already been characterized and reported elsewhere (McCormick et al. 2018, 2019). These studies showed that 2-stroke engines produced louder sounds overall, with higher average sound pressure levels (root-mean-square, ∼1.5 dB higher), louder maximum sounds (peak levels, ∼4 dB higher), stronger particle acceleration, and noise that lasted longer during boat passes (90% energy envelope). They also produced intense noise more consistently. In contrast, 4-stroke engines were slightly quieter but had greater acoustic complexity, and their noise was more discrete and shorter in duration. In addition to the two different boat noise treatments, there was also a control treatment, in which no boat was driven near the site and consisted of only naturally occurring ambient ocean noise (methods adapted from Harding et al. 2020). Each of the goby-shrimp burrows was allocated randomly to 1 of the 3 treatments, ensuring similar representation across all five sites (control, 4-stroke, or 2-stroke: *n* = 51, 40, and 51, respectively; see Table S1 for site-specific breakdown). The boat noise was implemented following protocols of Harding et al. (2020), with the 4-stroke or 2-stroke boat being driven using varying steering patterns for 10 min, 10 to 200 m from the focal burrow.

### Field experiments

Trials involved filming burrows of Steinitz's goby and snapping shrimp before, during, and after being exposed to one of the three noise treatments. Burrows were selected by first locating a large burrow opening, then confirming the presence of *A. steinitzi* (>10 cm SL) before proceeding. Once a suitable goby–shrimp pair had been identified, MMP positioned a video camera (GoPro Hero 5, GoPro Inc.) approximately 2 m from the burrow entrance and started the video recording before snorkeling away from the site. The experimental trial had four parts: a 20 min acclimation period when no behavior was measured, and then three distinct 10 min phases (Fig. 2). The first 10 min was the “pre” treatment phase, which was used to acquire a measure of baseline behavior and activity from the focal goby and shrimp. This was followed by a 10 min “during” treatment phase, when the noise treatments were administered (ie, control, 4-stroke, or 2-stroke). The final 10 min were the “post” treatment phase and were used to test potential carry-over effects after the noise exposure had ceased. Over the 4 weeks of the study, trials were conducted using a randomized combination of sites, noise treatments, and specific boats to minimize potential confounding effects from these factors. To avoid resampling the same gobies, each burrow's location was mapped. All trials were conducted between 14:00 and 17:00 h (*N* = 142).

### Behavioral measurements

For all response variables, only the middle 5 min (eg, between 2.5 to 7.5 min) for each of the three experimental phases (pre-, during-, and post-) were extracted for data analysis (Fig. 2). This was done to avoid any potential overlap of sound conditions at the junction of the different phases. The 5 min recordings were labeled with a code, their order randomized, and their audio removed (by RTM), so that the data extraction process, carried out by JLM, was blinded to treatment and phase.

All behavioral measurements were manually scored, with the keylogging behavioral analysis software BORIS v. 7.10.2 (Friard and Gamba 2016). The ethogram used to score behavior was designed by JMM and JLM, with input from all co-authors. To investigate the effects of boat noise pollution on risk perception, the total time gobies and shrimp spent refuging was recorded. The time spent refuging was defined as the total time spent partly or entirely inside the burrow (ie, having any body part inside the burrow was considered refuging). Refuging is a common antipredator behavior frequently used as a proxy of risk perception (Bonenfant and Kramer 1996; Wong et al. 2005; Polverino et al. 2024). Therefore, when there is a high perceived risk, individuals are expected to spend more time refuging (Lima and Dill 1990).

To assess if boat noise affects the communication between the mutualistic partners, the total time an individual spent in contact with a heterospecific partner was recorded. The goby communicates potential threats to the shrimp primarily through tactile signals (Preston 1978; Kingston et al. 2019), such as fin flicks, which the shrimp detects via their elongated antennae (Karplus 1979). However, as antennal contact was often not possible to ascertain from the recordings, we adopted a conservative proxy for contact. Specifically, focal animals were deemed to be in contact with one another when the most anterior point of the shrimp's rostrum was within 1 body length of the goby or when both individuals were inside the burrow together (antennae are approximately 1.5× the body length of a shrimp; Karplus and Thompson 2012). All behavioral measurements were scored for the three phases (pre-, during, and post-treatment, see Fig. 2).

### Statistical analysis

Data were analyzed in *RStudio* (v. 2023.09.1, Posit Software, PBC) and *R* (v. 4.3.2, 2023, the R Foundation for Statistical Computing) by JLM, with input from JMM. From the original 142 burrows that were sampled, 19 did not contain at least one shrimp and one goby. These burrows were removed from analysis, leaving a total of 123 burrows (control: *n* = 39; 4-stroke: *n* = 37; 2-stroke: *n* = 47). Some burrows contained multiple gobies and/or shrimp, which prevented our ability to preserve individual identity. Consequently, behaviors were recorded for all individuals of each species (goby or shrimp), and the scores were averaged by the number of individuals from that species in each trial.

The time that gobies and shrimp spent refuging and in contact with one another was recorded as a proportion of the total trial time. Refuging behavior and contact were modeled using Bayesian generalized linear mixed-effects models with zero-one inflated beta distributions (*brms* package; Bürkner 2017). Traditional beta distributions cannot handle boundary values of 0 and 1; therefore, the zero-one inflated beta distribution was chosen to handle cases where the goby or shrimp spent the entire observation period either outside/inside the burrow or in contact/apart from one another, resulting in exact values of 0 or 1. The predictor variables included the noise treatment (control, 4-stroke, or 2-stroke), the phase of the experiment (pre-, during-, or post-noise exposure), and the interaction between noise treatment and phase. Covariates included the species of the shrimp, the number of shrimps, and the number of gobies in each burrow. Burrow ID, nested within sample site, was included as a random intercept to account for the repeated measures design (ie, each burrow was measured at the three distinct phases). The models included shrimp species to account for the fact that various species of snapping shrimp (*Alpheus* spp.) form these mutualisms within the Lizard Island lagoon, and that they may be differentially sensitive to noise pollution.

Each model was run across four chains using broadly noninformative priors for 4,000 iterations with 1,000 warm-ups. The convergence of the models was ascertained through adequate mixing observed in the trace plots (evidenced by R-hat values being 1). To incorporate all the coefficients associated with both the zero-one inflation process and the beta distribution within our model, the *emmeans* package (version 1.10.7; Lenth 2025) was used. This facilitated the calculation of the estimated marginal mean posterior distributions for each level of the fixed effects and their interactions. Furthermore, *emmeans* was used to compute pairwise contrasts between experimental phases (pre, during, and post) for each noise treatment. The model predictions are presented as estimated marginal means accompanied by 95% credible intervals (CrI), with inference based on contrast estimates where CrIs do not overlap with zero. For all models, the cumulative number of boat passages at each site prior to a burrow's use in the experiment was included as a covariate in a post hoc assessment of potential site-level carry-over effects. The inclusion of this covariate did not meaningfully improve model fit and was not predictive of behavioral responses (see Tables S2 and S3).

## Results

### Goby behavior

**Fig. 3.**
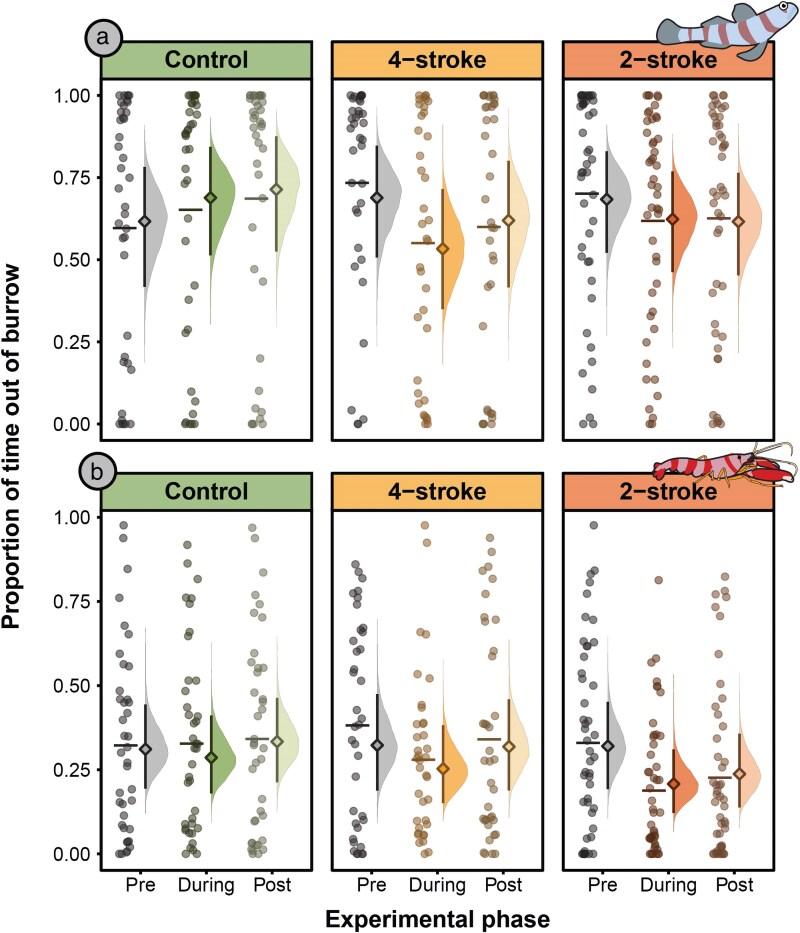
The proportion of time gobies spent outside their burrow (a) and shrimp spent outside their burrow (b), before (pre), during, and after (post) exposure to different noise treatments: control (*n* = 39), 4-stroke boat noise (*n* = 37), and 2-stroke boat noise (*n* = 47). The left side of each treatment by exposure phase, displays the raw data, with scatter plots and a horizontal line indicating the raw data mean. The right side displays model estimates, with the diamonds representing the model-estimated marginal mean, error bars the 95% CrI, and density plots the posterior distribution. For pairwise comparisons see Tables S5 and S6.

For control gobies, the proportion of time spent out of their burrows did not differ across the pre-, during-, and post-exposure phases of the experiment (all estimated marginal contrasts overlap with zero; Fig. 3a, Tables S4 and S5). Similarly, for gobies exposed to 2-stroke boat noise, there was no discernible change in the time spent outside their burrows across the three phases (Fig. 3a, Tables S4 and S5). However, for gobies subjected to the 4-stroke boat noise, there was a 22% reduction in the time they spent out of their burrows (Fig. 3a, Tables S4 and S5). Prior to noise exposure, the average proportion of time gobies spent outside of their burrows was 0.69 [0.51, 0.85, lower and upper 95% CrI; respectively], but during the 4-stroke boat noise, this decreased to 0.53 [0.35, 0.72], a mean reduction of 0.15 [0.01, 0.29]. Once the noise ceased in the post-exposure phase, goby burrow use returned to near pre-exposure levels (0.60 [0.41, 0.78]; Fig. 3a, Tables S4 and S5).

The number of gobies in the burrow had a marginal influence on the proportion of time gobies spent out of the burrow. Specifically, if only one goby was present (*n* = 28 cases), the average proportion of time that goby was out of the burrow was 0.58 [0.42, 0.73], whereas when two gobies were present (*n* = 95 cases), the average proportion of time each goby was out of the burrow was 0.71 [0.55, 0.84], a difference of 0.13 [0.00, 0.25] (however this difference was marginal, as the CrI's include 0; Table S5). Similarly, the number of shrimps in the burrow influenced the proportion of time that gobies spent out of the burrow. Specifically, when only one shrimp was present (*n* = 25 cases), the average proportion of time gobies spent out of the burrow was 0.57 [0.41, 0.71], whereas when two shrimps were present (*n* = 95 cases), this increased to 0.71 [0.61, 0.80], a difference of 0.14 [0.03, 0.27] (Table S5). In rare cases (*n* = 3), there were three shrimp in the burrow. However, because of the low occurrence, we have low confidence in these estimates. Moreover, contrasts comparing three shrimp to one or two shrimp revealed no meaningful differences (differences of 0.09 [−0.22, 0.36] and −0.06 [−0.37, 0.19], respectively). Lastly, the species of shrimp in the burrow did not affect the proportion of time gobies spent out of the burrow (Table S5).

### Shrimp behavior

For both the control shrimp and those exposed to 4-stroke boat noise, the proportion of time spent out of their burrows did not differ across the pre-, during-, and post-exposure phases of the experiment (Fig. 3b, Tables S4 and S6). Conversely, shrimp exposed to the 2-stroke boat noise exhibited a noticeable reduction in the proportion of time spent out of the burrow (Fig. 3b, Tables S4 and S6). Specifically, before exposure to the 2-stroke noise, shrimp spent on average 0.32 [0.19, 0.45] and during noise exposure they spent on average 0.21 [0.12, 0.31], a difference of 0.11 [0.03, 0.20], a 34% decrease in the amount of time they spent out of their burrow. This change in the proportion of time spent out of the burrow returned towards baseline in the post-exposure phase (0.24 [0.14, 0.36]), however, it should be noted that our effect estimates partially support a difference between the pre- and post-noise phases as the CrI overlapped marginally with zero (0.08 [−0.00, 0.17]; Fig. 3b, Tables S4 and S6).

The number of gobies and the species of shrimp did not affect the proportion of time the shrimp spent outside of their burrow (Table S6). The number of shrimp present did affect their time out of the burrow, with the average proportion of time spent out of their burrow increasing by 0.12 [0.02, 0.20] when two shrimp were present (*n* = 95 cases), compared to when one shrimp was present (*n* = 25 cases; Table S6). Again, in the few cases where three shrimp were present (*n* = 3), no change in burrow use was observed—likely due to the high uncertainty associated with such a low occurrence. Burrow use did not differ between the different species of shrimps (Table S6).

### Shrimp-goby communication

**Fig. 4.**
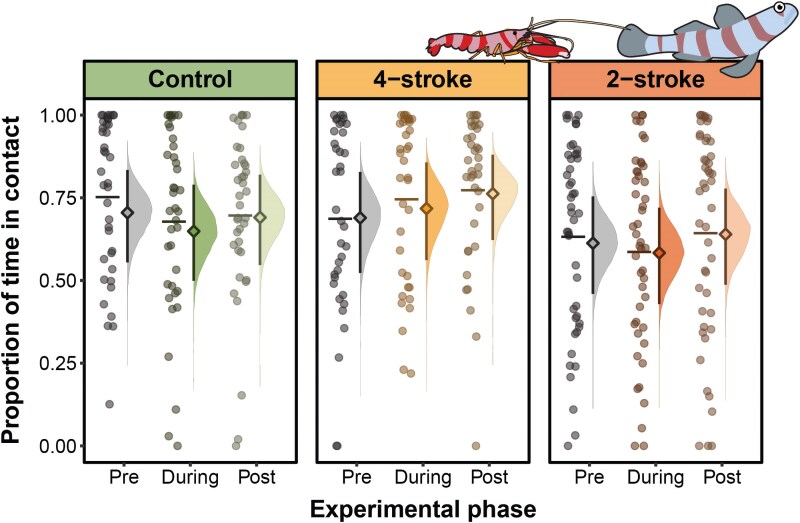
The proportion of time that gobies and shrimp were in contact before (pre), during, and after (post) exposure to different noise treatments: control (*n* = 39), 4-stroke boat noise (*n* = 37), and 2-stroke boat noise (*n* = 47). The left side of each treatment by exposure phase, displays the raw data, with scatter plots and a horizontal line indicating the raw data mean. The right side displays model estimates, with the diamonds representing the model-estimated marginal mean, error bars the 95% CrI, and density plots the posterior distribution. For pairwise comparisons, see Table S7.

The proportion of time shrimp maintained physical contact with a goby was unaffected by the different noise treatments (control, 4-stroke, and 2-stroke; Fig. 4, Tables S4 and S7), number of gobies, number of shrimp, and the species of shrimp (see Table S7). Overall, shrimp spent on average 0.67 [0.54, 0.79] of their time in contact with a goby.

## Discussion

Our results reveal that boat noise pollution can significantly alter the behavior of shrimps and gobies in a mutualistic partnership. Exposure to boat noise increased the tendency for both gobies and shrimp to seek refuge—a response that indicates an elevated perception of risk. Importantly, the data show that 2-stroke and 4-stroke boat noise elicit distinct behavioral responses. Shrimp displayed a pronounced reaction to 2-stroke engine noise, whereas gobies responded to 4-stroke engine noise. Despite these shifts in individual behaviors, the tactile communication for their mutualism was unaffected by noise exposure.

The overall increase in refuging behavior supports the hypothesis that both gobies and shrimp perceived the boat noise as an increased risk (2-stroke or 4-stroke, respectively). This aligns broadly with work on other refuge-seeking species’ (Jennions et al. 2003), including other species of gobies (Polverino et al. 2024), which show that animals adjust their burrow use to match perceived threat levels. The increase in perceived risk could arise from the gobies interpreting the noise itself as a direct threat or from the noise interfering with their ability to detect other threats, potentially via acoustic masking or cognitive impairment (Chan et al. 2010). Similar increases in refuging behavior in response to boat noise have been observed in other species, such as red-mouthed gobies (*Gobius cruentatus*; Picciulin et al. 2010) and Ward's damselfish (*Pomacentrus wardi*; McCormick et al. 2018). These changes in burrow use can be costly, as although seeking refuge can reduce predation risk, it also limits the opportunities for feeding, mating, and defending territories (Frid and Dill 2002). Such trade-offs can have significant fitness consequences; for instance, male Lusitanian toadfish (*Halobatrachus didactylus*) exposed to boat noise spent more time performing antipredator behaviors and less time courting potential mates, ultimately leading to reduced reproductive success (Amorim et al. 2022).

Our results reveal species-specific responses to the different engine types, each of which has distinct acoustic signatures. Gobies only exhibited a behavioral response to 4-stroke noise, which is characterized by a more complex sound (Jain-Schlaepfer et al. 2018; McCormick et al. 2018). In contrast, shrimp were affected by 2-stroke engine noise, which is associated with a higher overall intensity (McCormick et al. 2018). The differences in taxon responses may be partly explained by variation in auditory structures: snapping shrimp primarily detect sound using statocysts (Dinh and Radford 2021), whereas gobies predominantly use saccules (Vetter 2025). Although both organs are tuned to low frequencies—specifically the particle acceleration component of sound—studies in closely related species indicate that snapping shrimp have peak sensitivities around 80 to 100 Hz (*Alpheus richardsoni*; Dinh and Radford 2021) and gobies around 100 to 300 Hz (*Pomatoschistus pictus* and *P. marmoratus*; Amorim et al. 2018). Acoustic measurements by McCormick et al. (2018) show that 2-stroke engines emit disproportionately high particle acceleration noise at lower frequencies, which aligns more with shrimp hearing sensitivity and may explain their stronger response to 2-stroke noise.

With that said, this frequency-based explanation does not account for the gobies’ reaction to 4-stroke noise, which is relatively lower-intensity across their hearing range (∼100 to 300 Hz). This suggests that factors beyond frequency sensitivity—such as differences in the processing of acoustic signals—may underlie these species-specific responses. Similar patterns have been observed in other species: for example, 2-stroke engines were shown to elicit more pronounced stress responses in staghorn damselfish (*Amblyglyphidodon curacao*; Jain-Schlaepfer et al. 2018), whereas 4-stroke noise triggered stronger startle responses in juvenile whitetail damselfish (*Pomacentrus chrysurus*; McCormick et al. 2019). These species-specific sensitivities highlight the complexity of how different engine noises are processed and perceived and are reflective of broad taxa-level differences in responses to noise pollution (Kunc et al. 2016). Incorporating auditory-evoked potential measurements (changes in brain activity produced by auditory stimuli) could offer a more mechanistic insight into how these sounds are being perceived by the different species, and not just their relative intensities. Regardless of the mechanism, species-specific sensitivity to the different noise spectra may represent a wider challenge for management strategies aimed at reducing the impacts of aquatic noise pollution. Thus, selectively reducing a given engine type may not alleviate risks for all species and could, in fact, exacerbate impacts for some. Moreover, in real-world contexts, multiple boats with different engine types operate simultaneously, creating more complex soundscapes than tested here. Such overlapping exposures could generate interactive or compounding effects, particularly for species interactions, with the potential to further disrupt ecological interactions. Exploring these combined scenarios will therefore be an important avenue for future research, to better align experimental designs with the multi-source nature of anthropogenic noise.

Importantly, our results do not indicate substantial carry-over effects on behavior, as the increased refuge use observed during noise exposure returned towards baseline once the noise ceased. This aligns with findings from other studies where behavioral alterations induced by noise exposure were short-lived (Bruintjes et al. 2016). However, carry-over effects have been documented in other species, such as increased aggression in orange-fin anemonefish (*Amphiprion chrysopterus*; Mills et al. 2020) and reduced cleaning efficiency in bluestreak cleaner wrasse (*Labroides dimidiatus*; Nedelec et al. 2017b). Taken together, our results and those of previous studies underscore the importance of species-specific investigations to fully understand the long-term impacts of noise pollution on marine ecosystems.

Despite the shifts in individual refuge use, boat noise exposure did not impact the tactile communication between shrimp and gobies, suggesting that this aspect of their mutualism is relatively resistant to noise disturbances. Typically, when gobies are outside the burrow entrance, but the shrimp is still inside, the former will maintain contact with the latter. As a result, even when gobies alter the amount of time they spend inside the burrow, physical contact with their partner is preserved. This sentinel-like positioning is a defining feature of goby–shrimp partnerships: gobies remain alert at the burrow entrance, while shrimp rely on tactile cues to assess safety before emerging. Because these tactile signals are central to their cooperative interactions (Karplus and Thompson 2012), their persistence despite noise exposure highlights the resilience of this communication system (Burns et al. 2019).

In conclusion, this study provides important evidence that boat noise pollution significantly alters the behavior of animals engaged in mutualistic partnerships. While the interspecies interaction between gobies and shrimp remained intact, their refuging behavior was affected by noise exposure. Notably, species exhibited differential sensitivity to 2-stroke and 4-stroke engine noise, highlighting the complexity of noise pollution effects. These findings emphasize the importance of field-based research in understanding the real-world impacts of anthropogenic noise on marine species and underscore the need for targeted conservation efforts to mitigate the increasing threat of noise pollution.

## Supplementary Material

araf110_Supplementary_Data

## Data Availability

Analyses reported in this article can be reproduced using the data provided by Manera et al. (2025).
