# Supplementary material for: Boat noise alters individual behaviors but not communication between partners in a fish-shrimp mutualism
Source: Behav Ecol. 2025 Sep 27;36(5):araf110. doi: 10.1093/beheco/araf110 (PMC12527286; doi:10.1093/beheco/araf110)
Supplement: araf110_Supplementary_Data [file araf110_supplementary_data.docx]

**SUPPORTING INFORMATION**

**Boat noise alters individual behaviours but not communication between partners in a fish-shrimp mutualism.**

The following Supporting Information is available for this article:

**Table S1** Site specific samples sizes within each treatment.

**Table S2** Comparison of cumulative-sum versus non-cumulative models using approximate leave-one-out cross-validation.

**Table S3** The effect of the number of prior boat-noise trials at a site on each behavioural measure.

**Table S4** The estimated marginal means for the different phases of each noise treatments on the time gobies and shrimp spent out of their burrow and in contact with each other.

**Table S5** Pairwise comparisons for the proportion of time gobies spent outside of the burrow.

**Table S6** Pairwise comparisons for the proportion of time shrimp spent outside of the burrow.

**Table S7** Pairwise comparisons for the proportion of time shrimp spent in contact with a goby

**Table S1.** The number of burrows sampled at each of the five sites in the Lizard Island Lagoon, Great Barrier Reef, Australia (14°41'9"S, 145°27'21"E) and the number of burrows allocated to each treatment.

| **Site** | **Control** | **4-stroke** | **2-stroke** | **Total** |
| --- | --- | --- | --- | --- |
| 1 | 11 | 8 | 8 | 27 |
| 2 | 12 | 8 | 11 | 31 |
| 3 | 10 | 8 | 8 | 26 |
| 4 | 10 | 8 | 12 | 30 |
| 5 | 8 | 8 | 12 | 28 |
| **Total** | 51 | 40 | 51 | 142 |

**Table S2.** Model comparison of cumulative-sum versus non-cumulative models for behavioural responses using approximate leave-one-out cross-validation (LOO). Shown are expected log predictive densities (elpd LOO ± *SE*), the corresponding LOO information criteria (LOOIC), and pairwise differences (elpd diff ± *SE*) between the models.

| **Behavioural measure** | **Model** | **elpd LOO** | ***SE***  **(elpd LOO)** | **LOOIC** | **elpd diff** | ***SE***  **(elpd diff)** |
| --- | --- | --- | --- | --- | --- | --- |
| Goby: time outside burrow | Non-cumulative model | -122.687 | 20.224 | 245.374 | 0.000 | 0.000 |
|  | Cumulative-sum model | -123.597 | 20.292 | 247.194 | -0.910 | 1.438 |
| Shrimp: time outside burrow | Non-cumulative model | -34.989 | 22.245 | 69.978 | 0.000 | 0.000 |
|  | Cumulative-sum model | -36.140 | 22.430 | 72.280 | -1.151 | 1.722 |
| Shrimp-goby contact | Cumulative-sum model | -26.005 | 23.309 | 52.010 | 0.000 | 0.000 |
|  | Non-cumulative model | -27.516 | 23.375 | 55.032 | -1.511 | 1.852 |

**Table S3.** Effect of the possible cumulative prior exposure to boat noise on behavioural responses. Estimated slopes (± *SE* and 95% credible intervals) describe the effect of the number of prior boat-noise trials at a site on each behavioural measure.

| **Behavioural response** | **Trend per additional prior boat trial** | **Lower 95% CrI** | **Upper 95% CrI** |
| --- | --- | --- | --- |
| Proportion of time gobies spent outside of the burrow | 0.025 | -0.009 | 0.058 |
| Proportion of time shrimp spent outside of the burrow | 0.008 | -0.020 | 0.037 |
| Proportion of time shrimp spent in contact with a goby | -0.014 | -0.046 | 0.018 |

**Table S4.** The estimated marginal means and accompanying 95% credible intervals for each combination of phase and noise exposure on the time gobies and shrimp spent out of the burrow and in contact with each other.

| **Behavioural response** | **Phase** | **Noise exposure** | **Estimated marginal mean** | **Lower 95% CrI** | **Upper 95% CrI** |
| --- | --- | --- | --- | --- | --- |
| Proportion of time gobies spent outside of the burrow | Pre | control | 0.617 | 0.417 | 0.782 |
|  | Pre | 4-stroke | 0.688 | 0.507 | 0.847 |
|  | Pre | 2-stroke | 0.684 | 0.521 | 0.830 |
|  | During | control | 0.688 | 0.513 | 0.843 |
|  | During | 4-stroke | 0.533 | 0.349 | 0.715 |
|  | During | 2-stroke | 0.623 | 0.462 | 0.769 |
|  | Post | control | 0.714 | 0.525 | 0.876 |
|  | Post | 4-stroke | 0.619 | 0.416 | 0.801 |
|  | Post | 2-stroke | 0.616 | 0.453 | 0.764 |
| Proportion of time shrimp spent outside of the burrow | Pre | control | 0.311 | 0.194 | 0.444 |
|  | Pre | 4-stroke | 0.331 | 0.193 | 0.486 |
|  | Pre | 2-stroke | 0.322 | 0.193 | 0.454 |
|  | During | control | 0.286 | 0.180 | 0.411 |
|  | During | 4-stroke | 0.259 | 0.155 | 0.392 |
|  | During | 2-stroke | 0.209 | 0.122 | 0.312 |
|  | Post | control | 0.333 | 0.212 | 0.463 |
|  | Post | 4-stroke | 0.326 | 0.193 | 0.471 |
|  | Post | 2-stroke | 0.239 | 0.139 | 0.359 |
| Proportion of time shrimp spent in contact with a goby | Pre | control | 0.705 | 0.555 | 0.834 |
|  | Pre | 4-stroke | 0.689 | 0.523 | 0.828 |
|  | Pre | 2-stroke | 0.613 | 0.459 | 0.754 |
|  | During | control | 0.649 | 0.499 | 0.790 |
|  | During | 4-stroke | 0.718 | 0.562 | 0.857 |
|  | During | 2-stroke | 0.583 | 0.429 | 0.720 |
|  | Post | control | 0.691 | 0.547 | 0.820 |
|  | Post | 4-stroke | 0.762 | 0.623 | 0.880 |
|  | Post | 2-stroke | 0.640 | 0.487 | 0.778 |

***Table S5.*** *Estimated marginal difference in the proportion of time gobies spent outside of the burrow for the pairwise comparisons between the different levels of the predictor variables.*

| **Predictor variable** | **Contrast** | **Estimated marginal difference** | **Lower 95% CrI** | **Upper 95% CrI** |
| --- | --- | --- | --- | --- |
| Treatment and phase interaction | 2-Stroke Pre - 2-Stroke During | 0.061 | -0.041 | 0.166 |
| Treatment and phase interaction | 2-Stroke Pre - 2-Stroke Post | 0.067 | -0.045 | 0.186 |
| Treatment and phase interaction | 4-Stroke Pre - 4-Stroke During | 0.152 | 0.013 | 0.286 |
| Treatment and phase interaction | 4-Stroke Pre - 4-Stroke Post | 0.067 | -0.081 | 0.222 |
| Treatment and phase interaction | 4-Stroke Pre - 2-Stroke Pre | 0.005 | -0.159 | 0.138 |
| Treatment and phase interaction | Control Pre - Control During | -0.069 | -0.206 | 0.063 |
| Treatment and phase interaction | Control Pre - Control Post | -0.092 | -0.254 | 0.057 |
| Treatment and phase interaction | Control Pre - 2-Stroke Pre | -0.067 | -0.227 | 0.088 |
| Treatment and phase interaction | Control Pre - 4-Stroke Pre | -0.071 | -0.255 | 0.107 |
| Number of gobies | 2 gobies - 1 goby | 0.128 | -0.001 | 0.249 |
| Number of shrimp | 2 shrimp - 1 shrimp | 0.143 | 0.025 | 0.269 |
| Number of shrimp | 3 shrimp - 1 shrimp | 0.087 | -0.218 | 0.355 |
| Number of shrimp | 3 shrimp - 2 shrimp | -0.057 | -0.366 | 0.187 |
| Shrimp species | *A. mannarensis - A. bellulus* | 0.095 | -0.112 | 0.293 |
| Shrimp species | *A. sciolii - A. bellulus* | 0.081 | -0.209 | 0.329 |
| Shrimp species | *A. sciolii - A. mannarensis* | -0.013 | -0.224 | 0.183 |
| Shrimp species | unknown *- A. bellulus* | 0.114 | -0.110 | 0.341 |
| Shrimp species | unknown *- A. mannarensis* | 0.018 | -0.114 | 0.145 |
| Shrimp species | unknown *- A. sciolii* | 0.031 | -0.180 | 0.255 |

**Table S6.** Estimated marginal difference in the proportion of time shrimp spent outside of the burrow for the pairwise comparisons between the different levels of the predictor variables.

| **Predictor variable** | **Contrast** | **Estimated marginal difference** | **Lower 95% CrI** | **Upper 95% CrI** |
| --- | --- | --- | --- | --- |
| Treatment and phase interaction | 2-Stroke Pre - 2-Stroke During | 0.111 | 0.030 | 0.195 |
| Treatment and phase interaction | 2-Stroke Pre - 2-Stroke Post | 0.082 | -0.003 | 0.166 |
| Treatment and phase interaction | 4-Stroke Pre - 4-Stroke During | 0.071 | -0.035 | 0.183 |
| Treatment and phase interaction | 4-Stroke Pre - 4-Stroke Post | 0.004 | -0.109 | 0.119 |
| Treatment and phase interaction | 4-Stroke Pre - 2-Stroke Pre | 0.008 | -0.109 | 0.142 |
| Treatment and phase interaction | Control Pre - Control During | 0.024 | -0.068 | 0.117 |
| Treatment and phase interaction | Control Pre - Control Post | -0.021 | -0.113 | 0.068 |
| Treatment and phase interaction | Control Pre - 2-Stroke Pre | -0.011 | -0.131 | 0.112 |
| Treatment and phase interaction | Control Pre - 4-Stroke Pre | -0.019 | -0.152 | 0.113 |
| Number of gobies | 2 gobies - 1 goby | 0.067 | -0.019 | 0.160 |
| Number of shrimp | 2 shrimp - 1 shrimp | 0.117 | 0.022 | 0.203 |
| Number of shrimp | 3 shrimp - 1 shrimp | 0.086 | -0.108 | 0.302 |
| Number of shrimp | 3 shrimp - 2 shrimp | -0.032 | -0.221 | 0.171 |
| Shrimp species | *A. mannarensis - A. bellulus* | 0.002 | -0.175 | 0.155 |
| Shrimp species | *A. sciolii - A. bellulus* | -0.099 | -0.302 | 0.090 |
| Shrimp species | *A. sciolii - A. mannarensis* | -0.103 | -0.221 | 0.039 |
| Shrimp species | unknown *- A. bellulus* | -0.011 | -0.205 | 0.160 |
| Shrimp species | unknown *- A. mannarensis* | -0.013 | -0.103 | 0.091 |
| Shrimp species | unknown *- A. sciolii* | 0.089 | -0.059 | 0.237 |

**Table S7.** Estimated marginal differences in the proportion of time shrimp spent in contact with a goby for the pairwise comparisons between the different levels of the predictor variables.

| **Predictor variable** | **Contrast** | **Estimated marginal difference** | **Lower 95% CrI** | **Upper 95% CrI** |
| --- | --- | --- | --- | --- |
| Treatment and phase interaction | 2-Stroke Pre - 2-Stroke During | 0.029 | -0.043 | 0.106 |
| Treatment and phase interaction | 2-Stroke Pre - 2-Stroke Post | -0.026 | -0.105 | 0.061 |
| Treatment and phase interaction | 4-Stroke Pre - 4-Stroke During | -0.028 | -0.128 | 0.072 |
| Treatment and phase interaction | 4-Stroke Pre - 4-Stroke Post | -0.071 | -0.163 | 0.016 |
| Treatment and phase interaction | 4-Stroke Pre - 2-Stroke Pre | 0.075 | -0.045 | 0.197 |
| Treatment and phase interaction | Control Pre - Control During | 0.054 | -0.039 | 0.156 |
| Treatment and phase interaction | Control Pre - Control Post | 0.013 | -0.080 | 0.106 |
| Treatment and phase interaction | Control Pre - 2-Stroke Pre | 0.091 | -0.030 | 0.215 |
| Treatment and phase interaction | Control Pre - 4-Stroke Pre | 0.015 | -0.113 | 0.147 |
| Number of gobies | 2 gobies - 1 goby | 0.015 | -0.085 | 0.109 |
| Number of shrimp | 2 shrimp - 1 shrimp | -0.053 | -0.152 | 0.042 |
| Number of shrimp | 3 shrimp - 1 shrimp | -0.087 | -0.336 | 0.133 |
| Number of shrimp | 3 shrimp - 2 shrimp | -0.034 | -0.270 | 0.174 |
| Shrimp species | *A. mannarensis - A. bellulus* | 0.012 | -0.147 | 0.186 |
| Shrimp species | *A. sciolii - A. bellulus* | -0.149 | -0.377 | 0.084 |
| Shrimp species | *A. sciolii - A. mannarensis* | -0.163 | -0.343 | 0.011 |
| Shrimp species | unknown *- A. bellulus* | -0.050 | -0.235 | 0.143 |
| Shrimp species | unknown *- A. mannarensis* | -0.062 | -0.170 | 0.034 |
| Shrimp species | unknown *- A. sciolii* | 0.100 | -0.080 | 0.297 |
